# Angiotensin I-Converting Enzyme (ACE) Inhibitory Activity, Antioxidant Properties, Phenolic Content and Amino Acid Profiles of *Fucus spiralis* L. Protein Hydrolysate Fractions

**DOI:** 10.3390/md15100311

**Published:** 2017-10-13

**Authors:** Lisete Paiva, Elisabete Lima, Ana Isabel Neto, José Baptista

**Affiliations:** 1 Biotechnology Centre of Azores (CBA), University of Azores, 9501-801 Ponta Delgada, Portugal; lisete.s.paiva@uac.pt (L.P.); jose.ab.baptista@uac.pt (J.B.); 2 Research Center for Agricultural Technology (CITA-A), University of Azores, 9501-801 Ponta Delgada, Portugal; 3 Azorean Biodiversity Group, Centre for Ecology, Evolution and Environmental Changes (CE3C), Department of Biology, University of Azores, 9501-801 Ponta Delgada, Portugal; ana.im.neto@uac.pt

**Keywords:** edible brown algae, protein enzymatic hydrolysate, ultrafiltration, ACE-inhibition, antioxidant properties, phlorotannins, peptide fractions, amino acids composition, marine functional foods, cardiovascular-health

## Abstract

Food protein-derived hydrolysates with multi-bioactivities such as antihypertensive and antioxidant properties have recently received special attention since both activities can play significant roles in preventing cardiovascular diseases. This study reports, for the first time, the angiotensin I-converting enzyme (ACE)-inhibition and antioxidant properties of ultrafiltrate fractions (UF) with different molecular weight ranges (<1, 1–3 and ≥3 kDa) obtained from *Fucus spiralis* protein hydrolysate (FSPH) digested with cellulase–bromelain. The amino acids profile, recovery yield, protein, peptide and total phenolic contents of these FSPH-UF, and the in vitro digestibility of *F. spiralis* crude protein were also investigated. FSPH-UF ≥3 kDa presented remarkably higher ACE-inhibition, yield, peptide and polyphenolic (phlorotannins) contents. Antioxidant analysis showed that FSPH-UF <1 kDa and ≥3 kDa exhibited significantly higher scavenging of 2,2-diphenyl-1-picrylhydrazyl radical and ferrous ion-chelating (FIC) activity. FSPH-UF ≥3 kDa had also notably higher ferric reducing antioxidant power (FRAP). Strong correlations were observed between ACE-inhibition and antioxidant activities (FIC and FRAP). The results suggest that ACE-inhibition and antioxidant properties of FSPH-UF may be due to the bioactive peptides and polyphenols released during the enzymatic hydrolysis. In conclusion, this study shows the potential use of defined size FSPH-UF for the prevention/treatment of hypertension and/or oxidative stress-related diseases.

## 1. Introduction

Marine organisms, including macro-algae, are a valuable source of structurally diverse bioactive metabolites with various biological activities due to their living mode in highly competitive and aggressive surroundings, which are very different in many aspects from terrestrial environment, a situation that demands the production of quite specific and potent bioactive molecules. As a result, the importance of these organisms as a natural resource of bioactive compounds, which may lead to the development of new drugs and functional foods or nutraceuticals, is growing rapidly [1].

Macro-algae have been consumed in Asian countries since ancient times and their dietary intake has been shown to decrease blood pressure in humans [2]. However, the angiotensin I-converting enzyme (ACE) inhibitory activity from macro-algae has not been extensively studied [3]. ACE belongs to the class of zinc metal proteases that catalyzes the conversion of angiotensin I to a potent vasoconstrictor angiotensin II and also promotes the degradation of the vasodilator bradykinin [4]. Therefore, this multifunctional enzyme plays a key role in the control of blood pressure, since the inhibition of its activity leads to a decrease in the angiotensin II concentration and an increase in the bradykinin level, and consequently in the reduction of hypertension that is one of the major causes of chronic diseases and a high risk factor of cardiovascular diseases worldwide [5]. A wide variety of synthetic drugs have been extensively used in treatment of hypertension and most of them have an ACE-inhibitory activity, however, these drugs can cause certain adverse side effects [6]. Therefore, searching for ACE-inhibitors from natural resources, such as marine organisms including macro-algae [3,7,8,9], has shown a growing interest in the field of nutraceutical, pharmaceutical and functional foods industries. The most commonly studied natural ACE-inhibitors were protein hydrolysates and peptides, however, others molecules that affect the ACE activity include the phorotannins group that are the predominant polyphenols in brown algae, being particularly abundant in Fucaceae [10,11,12,13]. In addition to its strong ACE-inhibition, phlorotannins have been recently demonstrated to possess numerous health benefits, including potent antioxidant effects (for a review see [14]). Since functional food products with multi-bioactivities are receiving wider attention, the potential antioxidant of macro-algal protein hydrolysates/peptides with ACE-inhibitory activity has also been investigated [15,16,17]. In fact, it is well known that natural antioxidants are powerful substances that play an important role against various diseases (atherosclerosis, cancer, chronic inflammation, cardiovascular disorders, hypertension) and ageing process, directly related to oxidative stress [18].

There is substantial scientific evidence that enzymatic hydrolysis of food protein sources is an efficient method to recover potent bioactive peptides that may present lower side effects [1,19]. Furthermore, enzymatic proteolysis can also release other bioactive compounds such as phorotannins bound to proteins, as reported for some brown macro-algae [20].

The Azores Islands (Portugal) being isolated in the middle of Atlantic Ocean and surrounded by waters with low pollution levels [21] is a very promising location to look for new marine ingredients with medicine-like effects in treating or preventing chronic diseases. Traditionally, the Azorean population has gathered seaweeds either as food or for chemicals extraction. The brown seaweed *Fucus spiralis*, which is abundant in the Azorean intertidal zone, is a local delicacy, particularly the frond tips (the receptacles) that are picked and eaten fresh [22]. Previous studies on its nutritional and/or pharmacological value from our research group have reported that *F. spiralis* is a good source of valuable biochemical compounds [23] and its methanol extract could also be a source of powerful ACE-inhibitory phlorotannins with potential impact on human health [9]. *F. spiralis* from other origins have been also demonstrated to possess high antioxidant properties, mainly linked to its phlorotannin content [24,25,26].

This is, for the best of our knowledge, the first study of *F. spiralis* enzymatic protein hydrolysate as source of compounds with multi-bioactivities for potential use in food and pharmaceutical industries and it was aimed to: (i) optimize the protein extraction process to obtain higher yield of *F. spiralis* protein hydrolysate, (ii) fractionate the cellulase–bromelain protein hydrolysate by ultrafiltration membranes into different molecular weight fractions (<1, 1–3 and ≥3 kDa), (iii) evaluate the ultrafiltrate fractions for multifunctional properties in vitro, namely the ACE-inhibitory activity and the antioxidant properties using different assays, such as the scavenging of 2,2-diphenyl-1-picrylhydrazyl radical, the ferrous ion-chelating and the ferric reducing antioxidant power, (iv) determine the amino acids profile, recovery yield, protein, peptide and total phenolic contents of the ultrafiltrate fractions, and (v) evaluate the in vitro digestibility of *F. spiralis* crude protein. The findings of the current study can also contribute to the increasing database of medicinal macro-algae.

## 2. Results and Discussion

### 2.1. F. spiralis Protein Content and In Vitro Protein Digestibility Evaluation

Results revealed that *F. spiralis* has a protein content of 8.53 ± 0.03% (on a dry basis), which is within the range reported for brown algae (3.0–15.0%) [27]. *F. spiralis* protein digestibility, expressed as a relative percentage of sodium caseinate digestibility normalized at 100%, was 85.95 ± 0.85%. It is well known that amino acid profiles are important in evaluating the proteins quality, and their digestibility is the primary determinant of its amino acid availability, but the in vivo algae protein digestibility is still poorly described. However, the high protein digestibility determined in vitro suggests that *F. spiralis* can be used as a complementary source of food proteins for human and animal nutrition, which according to Fleurence [27], would be a promising way for the exploitation of marine resources in Europe.

### 2.2. Preparation of F. spiralis Protein Hydrolysate (FSPH) and Its Fractionation by Ultrafiltration

In this study, *F. spiralis* protein concentrate after a two steps hydrolysis with a combination of two enzymes (cellulase and bromelain) followed by membrane ultrafiltration yielded three fractions (Fr_1_ < 1 kDa, 1 kDa ≤ Fr_2_ < 3 kDa and Fr_3_ ≥ 3 kDa) that presented values of 206.28, 53.72 and 703.95 mg/g of dry FSPH, respectively (Table 1). These fractions were assessed for their ACE-inhibitory and antioxidant activities in order to find new marine sources of functional food products with multi-bioactivities for application in food and medicine industries. Indeed, according to some researchers [17,20], enzymatic hydrolysis can enhance the release of ACE inhibitory and antioxidant compounds (such as peptides and polyphenols) from the algal matrix. On the other hand, the use of carbohydrases, such as cellulase that breakdown cellulose and some related polysaccharides [28], can be useful before proteolytic enzymes applications. In fact the results showed that, when cellulase was used before the enzymatic hydrolysis with bromelain, the yield of *F. spiralis* protein concentrate was 1.5-fold increased. The choice of bromelain was based on our previous study [29] that showed the higher proteolytic activity (number of peptides) of bromelain as compared to the other nine commercial proteases tested using *F. spiralis* water extracts.

A two-steps hydrolysis was been previously used by our research group on other macro-algae proteins, to promote the release of low-MW (molecular weight) peptides [3]. Furthermore, this previous report also revealed that bromelain exhibited the highest specificity in the generation of ACE-inhibitory peptides from *Ulva rigida* protein. Other studies on other marine resources, such as different kinds of fish, also showed that bromelain is the most efficient protease tested for the production of protein hydrolysates with higher bioactivities (ACE-inhibitory and/or antioxidant activities) [30,31,32]. On the other hand, a molecular weight cut-off (MWCO) dialysis of FSPH was further used as means of enhancing its bioactivities, as well as to provide insights into the molecular weight distribution of the bioactive compounds presented in the hydrolysate. Furthermore, it should also be pointed out that the use of bioactive MWCO fractions instead of its purified active constituents for the development of functional foods presents multiple advantages. In fact, besides its low cost production and high production yield, MWCO fractions are formed by sets of compounds which could exhibit different biological activities and may act in synergy to increase its physiological effects on the organism [33]. In addition, the incorporation of protein hydrolysate fractions to foods could confer low cost desirable nutritional and functional properties.

### 2.3. Protein and Peptide Contents of FSPH Fractions

Protein and peptide contents of FSPH ultrafiltration fractions are also shown in Table 1, being the higher values in Fr_3_ (474.03 ± 4.44 and 243.82 ± 2.91 mg/g of dry FSPH, respectively). The Fr_1_ and Fr_2_ presented also a higher value of protein (123.15 ± 2.78 and 336.28 ± 4.96 mg/g of dry FSPH, respectively), but only a small content of peptides (43.81 ± 2.27 and 35.91 ± 1.58 mg/g of dry FSPH, respectively) as compared to Fr_3_.

### 2.4. Amino Acids Composition of FSPH Fractions

It is known that food protein hydrolysate/fractions containing appropriate amounts of amino acids with strong contribution properties could be potential candidates for use as potent antihypertensive and antioxidant agents [34]. As shown in Table 2, the amino acid’s profiles varied among the FSPH ultrafiltration fractions in terms of individual and total amino acid contents. The total amino acid content in Fr_3_ was 562.75 mg/g of dry FSPH which is significantly higher than those found in Fr_1_ and Fr_2_ (196.28 mg/g and 242.48 mg/g of dry FSPH, respectively). All the FSPH fractions showed remarkably higher percentage of the hydrophobic amino acids group (65.4–90.1% of the total amino acids), being valine the major amino acid in all fractions with 79.2%, 86.3% and 42.1% of the total amino acids in Fr_1_, Fr_2_ and Fr_3_, respectively. Glutamic acid, tyrosine, aspartic acid and isoleucine were the other major amino acids in Fr_3_, representing 8.2%, 6.8%, 6.7% and 5.8% of the total amino acids, respectively. In fractions Fr_1_ and Fr_2_, lysine and glutamic acid were the other major amino acids present. The amino acids content of Fr_1_ was very similar to that of Fr_2_. Overall, the Fr_3_ had the highest concentration of aromatic amino acids as well as hydrophobic aliphatic amino acids such as isoleucine, leucine, alanine, methionine and proline that, according to the previous reports [17,33,34], may promote the ACE-inhibitory and antioxidant activities of food protein hydrolysates/fractions.

According to the literature, most seaweeds contains all essential amino acids and some also present high levels of acidic amino acids (8.0–44.0% on a dry basis) and low levels of histidine, threonine, tryptophan, lysine, methionine and cysteine [35]. In our study methionine, threonine and lysine, in which many cereals are deficient [36], were presented in reasonable percentages, particularly in Fr_3_ and in Fr_1_.

### 2.5. Total Phenolic Content (TPC) of FSPH Fractions

**Figure 1:**
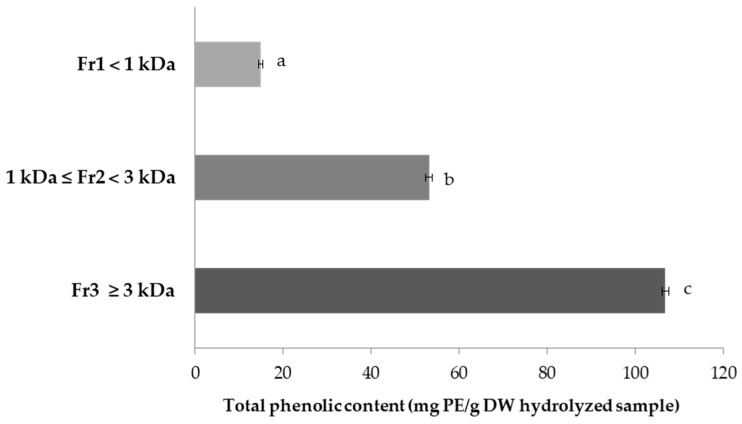
Total phenolic contents (TPC) of *F. spiralis* protein hydrolysate fractions obtained by ultrafiltration (tested concentration 2 mg/mL). Values are mean ± SD (*n =* 3). Different letters are significantly different (*p <* 0.05). Legend: DW, dry weight; PE, phloroglucinol equivalents.

The TPC was quantified as phloroglucinol equivalents (PE), and the results (Figure 1) revealed that Fr_3_ was the one with remarkably higher values (106.83 ± 0.76 mg PE/g of DW) followed by Fr_2_ and Fr_1_ with the values of 53.17 ± 0.76 and 15.00 ± 0.50 mg PE/g of DW, respectively, suggesting that high-MW phlorotannins are the largest pool of phenolic compounds in *F. spiralis*. On the other hand, the same order was observed for the protein content of FSPH fractions, as previously mentioned, suggesting that the fraction having a high content of protein tended to have a higher TPC. These findings are in accordance with Athukorala and Jeon [37] for the Flavourzyme *Ecklonia cava* hydrolysate fractions.

The TPC values of *F. spiralis* in the present study were higher than the ones reported by Tierney et al. [38] for Irish *F. spiralis*. This fact may be related with differences in the extraction methodology employed. Furthermore, significant intra-species variations of TPC in brown fucoid algae are well documented, showing the effect of several factors such as algae size, age, tissue type and environmental factors, including nutrient availability, light intensity, ultraviolet radiation, salinity, water depth and season [14,39].

According to the literature, brown macro-algae are a well-known source of phlorotannins, a structurally unique polyphenols that have gathered much attention due to their numerous bioactivities with high commercial interest for pharmaceutical, nutraceutical, cosmetic and especially food industries. Phlorotannins, which have been reported as the only phenolic group detected in some brown algae, such as *Fucus vesiculosus* [14], are highly hydrophilic components with a wide range of molecular sizes ranging between 126 Da and 650 kDa, although they are more commonly found in the 10 to 100 kDa range, particularly in the Fucaceae family [11,25]. Only few reports have focused on phlorotannin profile from *Fucus* spp. Phlorotannins with high degree of polymerization were observed in *F. spiralis* from Canada [40]; this information is important because it is known that ACE-inhibitory and antioxidant activities may depend on the degree of polymerization of phlorotannin derivatives [11,25]. Furthermore, sparse characterization of individual phlorotannin components has been carried out in *F. spiralis* species [9,24,41].

### 2.6. ACE-Inhibitory Activity of FSPH Fractions

The ACE-inhibition was determined by HPLC-UV method and the results (Table 1) revealed a remarkably higher activity in Fr_3_ followed by Fr_1_ and Fr_2_ with percentage values of 86.85 ± 1.89%, 45.08 ± 0.66% and 41.93 ± 1.62% and IC_50_ values of 0.500 ± 0.03, 1.850 ± 0.06 and 2.000 ± 0.06 mg/mL, respectively. However, all FSPH fractions showed higher IC_50_ values as compared to captopril (0.163 ng/mL), a synthetic ACE-inhibitor used in this study as a positive control. A comparison of the present data with other results is quite difficult due to the lack of literature on ACE-inhibition of *Fucus* spp. protein hydrolysates/fractions, as well as variations in hydrolysis conditions. However, the ACE IC_50_ values obtained in this study (0.500–2.000 mg/mL) were similar to those reported for bromelain–alcalase protein hydrolysate fractions from the sea cucumber *Acaudina molpadioidea* (0.615–1.975 mg/mL) [42]. Furthermore, our values were similar or lower than those described for *Porphyra yezoensis* hydrolysates (1.520–3.210 mg/mL) [7], and are within the range reported for enzymatic hydrolysates from different protein sources (0.20–2.47 mg/mL) with antihypertensive activity in spontaneously hypertensive rats [43].

The difference in the ACE-inhibition among hydrolysate fractions could be related to the differences in amino acid compositions (Table 2) and their sequences, as well as the peptide sizes. Indeed, ACE-inhibitory peptides are usually between 2 and 30 amino acids in size [44]. On the other hand, according to the literature [17,45,46], the presence of aromatic and branched-side chain amino acids (BCAA) in peptides may promote the ACE-inhibition of food protein hydrolysates/fractions. Thus, the results of this study suggested that the relatively good ACE-inhibition (over 40%) of the low-MW FSPH fractions (Fr_1_ and Fr_2_) can be attributed to the presence of low-MW peptides plus the highest concentration of BCAA. In turn, the Fr_3_ was the one with significantly higher aromatic amino acids content that make a substantial contribution to its ACE-inhibition by blocking angiotensin II production, probably due to their high steric properties and low lipophilicity [45]. Furthermore, the remarkably higher ACE-inhibition of Fr_3_ could also be due to the contribution of polyphenols (phlorotannins) released during the enzymatic hydrolysis and concentrated in this fraction. In fact, some reports indicate that brown algae might have strong ACE-like inhibitors associated not only with peptides but also with phlorotannins [11,12]. Wijesinghe et al. [12] also reported that the ACE-inhibition may be closely associated with protein-binding abilities of phlorotannins, which are characteristic of all tannins. Indeed, it has been well described that tannins have the ability to form strong complexes with proteins, either reversibly by hydrogen bonding through peptide or amide linkages or irreversibly by covalent condensation, and that polyphenolic compounds inhibit ACE activity through sequestration of the enzyme metal factor (Zn^2+^) [47]. A previous study by our research group [9] already reported that the strong ACE-inhibition by Azorean *F. spiralis* methanol extract may be due to its rich content of high-MW phorotannins, also concentrated in the MWCO ≥3 kDa fraction. Thus, *F. spiralis* phorotannins could be considered as potential phytopharmaceuticals for the development of new ACE inhibitors. On the other hand, it should be also pointed out that for food industry, enzymatic extracts, as obtained in the present study, is safer for consumers than organic solvent extracts, since the enzymatic method does not utilizes any organic solvent or other toxic chemicals.

### 2.7. Antioxidant Activities of FSPH Fractions

#### 2.7.1. 2,2-Diphenyl-1-picrylhydrazyl (DPPH) Free Radical Scavenging Activity (FRSA) Assay

**Figure 2:**
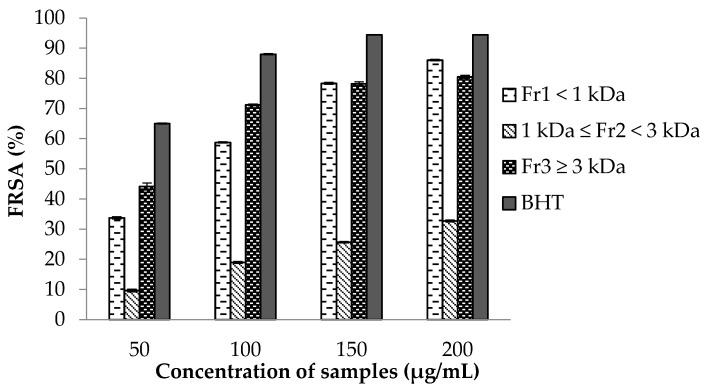
Free radical scavenging activity (FRSA) of *F. spiralis* protein hydrolysate fractions obtained by ultrafiltration. BHT (butylated hydroxytoluene) was used as positive control. Values are mean ± SD (*n =* 3).

The scavenging activity of DPPH free radicals had been used extensively to determine the antioxidant power of bioactive natural products. The results revealed that FRSA increased with increasing FSPH fraction concentration (Figure 2). At the highest concentration of 200 µg/mL the Fr_1_ presented the highest FRSA value (86.03 ± 0.25%), followed by Fr_3_ (80.50 ± 0.53%) whereas the Fr_2_ presented the lowest value (32.73 ± 0.32%); furthermore, the FRSA of Fr_1_ was comparable to that of the commercial antioxidant butylated hydroxytoluene (BHT). Tierney et al. [38] reported a lower value (6.18 ± 2.16%, the reciprocal of the IC_50_) of FRSA for the fraction with a MW of 3 kDa obtained from Irish *F. spiralis* hydrolysate digested with carbohydrase Viscozyme. These results may suggest that the enzymatic method used in the present study was more effective for the generation of antioxidant hydrolysates from *F. spiralis*. Several studies have also shown that, generally, low-MW hydrolysate fractions have higher DPPH radical scavenging activity than in high-MW hydrolysate fractions [48,49]. However, Fr_3_ showed an IC_50_ value of 55 µg/mL that is lower than Fr_1_ (85 µg/mL). Similar results were reported by Udenigwe et al. [50], who found that the high-MW fractions of flaxseed protein hydrolysate showed better FRSA as compared to the low-MW fractions.

The difference in the FRSA among the hydrolysate fractions might be related not only to their different peptide sizes but also to the differences in the amino acid compositions (Table 2) and their sequences, as well as the synergistic contributions of the active compounds. Indeed, research by Chen et al. [51] and Guo et al. [52] has demonstrated that DPPH radical scavenging activity is related to the amino acids composition. It was reported that aromatic amino acids and hydrophobic aliphatic amino acids (such as alanine, leucine, methionine and valine) play an important role in the DPPH radical scavenging activity [53,54]. Alemán et al. [55] also reports that the presence of those amino acids in a peptide sequence might increase the ability to access and neutralize the reactive free radicals and consequently to enhance the antioxidant activity. In addition, acidic amino acids had showed strong positive effects on scavenging of DPPH and, in contrast, positively-charged amino acids strongly contributed negatively to FRSA [34]. Thus, the lower FRSA IC_50_ value in Fr_3_ might be related to the highest content of aromatic, acidic and hydrophobic aliphatic amino acid residues, with exception for valine, as well as to the lowest positively-charged amino acids content. However, the superior TPC of Fr_3_ than in low-MW fractions (Fr_1_ and Fr_2_), as previously mentioned, may suggest that high-MW phlorotannins are the principal contributor to the lower FRSA IC_50_ value observed. Similar results were reported by other researchers, who found that high-MWCO fractions of *Fucus* species, such as *F. vesiculosus* [39] and *F. serrata* [56], had the highest FRSA and phenolic content (phlorotannins). It should also be highlighted that a recent study [26] revealed that phlorotannin-enriched fractions from *F. spiralis* from Peniche (Portugal) have the capacity to inhibit in vitro human cellular damage promoted by reactive oxygen species. Kim et al. [57] also reported that the fraction >30 kDa of the Celluclast enzymatic extract from brown algae *Ecklonia cava* possesses high protective effects against hydrogen peroxide-induced cell damage, attributed to the presence of phlorotannins. As in the present study, this *E. cava* fraction revealed the highest TPC, whereas the lowest content was shown by the fraction <1 kDa.

#### 2.7.2. Ferrous Ion-Chelating (FIC) Activity Assay

**Figure 3:**
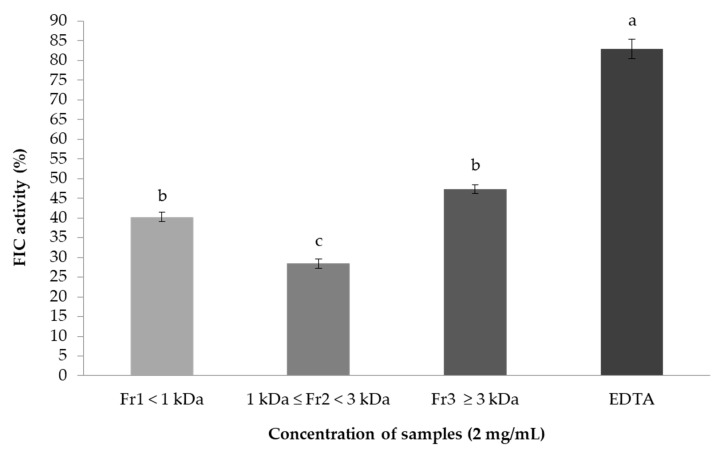
Ferrous ion-chelating (FIC) activities of *F. spiralis* protein hydrolysate fractions obtained by ultrafiltration. EDTA (ethylenediaminetetraacetic acid) was used as positive control. Values are mean ± SD (*n =* 3). Different letters are significantly different (*p <* 0.05).

Since metal chelating capacity is claimed as one of the important mechanisms of antioxidant activity [58], FIC assay was also chosen to better characterize the antioxidant activity of FSPH fractions. As shown in Figure 3, and in accordance with FRSA results, Fr_3_ presented the highest FIC activity (47.33 ± 1.15%) followed by Fr_1_ (40.28 ± 1.21%), and Fr_2_ the lowest value (28.47 ± 1.21%), but no significant difference was observed between Fr_1_ and Fr_3_. Similar results were found by Tierney et al. [38] for FIC activity (50.67 ± 5.58%) in the fraction with MW of 3 kDa from Irish *F. spiralis* Viscozyme hydrolysate. The FSPH Fr_1_ and Fr_3_ were moderate metal chelating agents as compared to the synthetic antioxidant EDTA, a potent metal-ion chelator used in this study as a positive control.

Most of the reported peptides exhibiting chelating activities were those with low-MW [59], which explains the FIC activity of the lower-MW Fr_1_. However, higher FIC activity in some other food hydrolysate fractions is linked to a larger peptide size [60]. Furthermore, peptides with high chelating activities have a high content of acidic amino acids [17]. As shown in Table 2, the Fr_3_ had the highest content of negatively charged amino acids followed by Fr_1_. On the other hand, Fr_3_ presented the highest TPC value, as previously mentioned. Thus, the highest FIC activity of the higher-MW Fr_3_ can be attributed to larger size bioactive compounds, such as the large peptides and the high-MW phlorotannins, released during the enzymatic hydrolysis and concentrated in this fraction. Indeed, some studies have demonstrated that polyphenols derived from algae are potent ferrous-ion chelators [61] and that its metal chelating potency is dependent upon their unique phenolic structure and the number and location of the hydroxyl groups [62]. However, to better evaluate the contribution of the polyphenols to the FIC activity of *F. spiralis*, additional research is needed on structural information of the bioactive compounds.

#### 2.7.3. Ferric Reducing Antioxidant Power (FRAP) Assay

**Figure 4:**
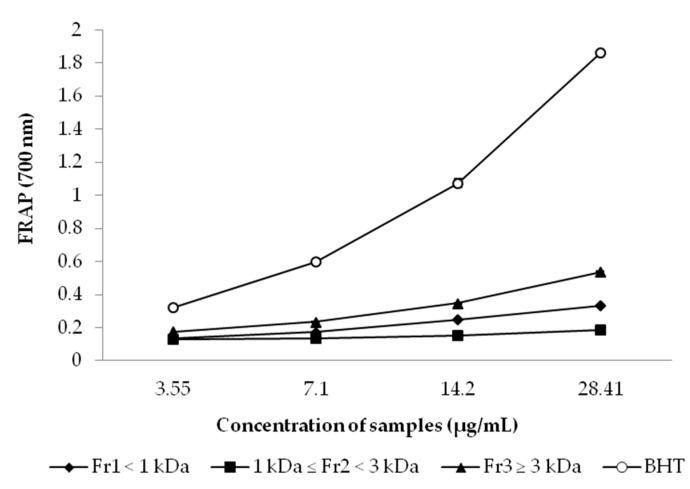
Ferric reducing antioxidant power (FRAP) of *F. spiralis* protein hydrolysate fractions obtained by ultrafiltration. BHT (butylated hydroxytoluene) was used as positive control. Values are mean ± SD (*n =* 3).

The highest FRAP was observed in Fr_3_ followed by Fr_1_ and Fr_2_ fractions (Figure 4) for the higher concentration used (28.41 µg/mL), presenting absorbance values of 0.538, 0.334 and 0.185, respectively. The presence of reducers (antioxidants) causes the conversion of the Fe^3+^/ferricyanide complex used in this method to the ferrous form. Therefore, by measuring the formation of Perl’s Prussian blue at 700 nm, we can monitor the Fe^2+^ concentration, that indicates a higher reducing power, compared to that of BHT, which is known to be a strong reducing agent. The information on FRAP activity is important because the reducing capacity of a compound/fraction may serve as a significant indicator of reductones, that are reported to be terminators of free radicals chain reaction, presents in the samples [63,64,65].

In accordance to the outlined for FRSA and FIC activity results, the highest FRAP of Fr_3_ may be due, mainly, to its rich content of high-MW phlorotannins. Our findings are in agreement with previous studies on FRAP of MW-fractionated hydrolysates/extracts from other algae samples, such as the Irish *F. spiralis* [25,38] and other *Fucus* species (*F. vesiculosus* and *F. serrata*) [39,56]. Furthermore, as shown in Table 2, Fr_3_ presented the highest content of the amino acids glutamic acid, aspartic acid, glycine, methionine and threonine as well as lysine in lower amount that, based on Udenigwe and Aluko [34], is a type of pattern that could have contributed to the highest FRAP observed in this FSPH fraction. Moreover, other authors observed that higher FRAP in some other food hydrolysate fractions is also linked to a larger size peptide content [60], that is in agreement with our results.

### 2.8. Pearson Correlation between Parameters

A significant correlation was found to occur among the methods for the antioxidant activities determination in all FSPH fractions (Table 3). FRSA and FIC activity (*r* = 0.890) were strongly correlated as well as FIC activity and FRAP (*r* = 0.973). A moderate correlation was found between FRSA and FRAP (*r* = 0.760). Concerning the correlation between TPC and antioxidant activities, a very weak correlation exists between TPC and FRSA (*r* = 0.003), and a weak correlation between TPC and FIC activity (*r* = 0.458), however, TPC and FRAP were moderately correlated (*r* = 0.652). On the other hand, strong correlations were observed between peptide content and FIC activity or FRAP or TPC (*r* = 0.805, *r* = 0.921 and *r* = 0.896), while the correlation between peptide content and FRSA was weak (*r* = 0.446). These results may reveal that other bioactive compounds such as peptides, in addition to polyphenols (phlorotannins), are contributing to the antioxidant activities of FSPH fractions evaluated by FRSA, FIC activity and FRAP assays. Previous research by Zubia et al. [66] and Vinayak et al. [67] also reported weak correlations (*r* = −0.399 and *r* = 0.397, respectively) between TPC and FRSA of macro-algal extracts. However, it should be noted that individual polyphenols may have a considerable antioxidant potential, but there may be antagonistic or synergistic interactions between phenolic and non-phenolic compounds that may affect the bioactivity [68]. Concerning the FIC activity, the correlations observed in the present study may suggest that polyphenols (phlorotannins) are not the principal chelators in some of FSPH fractions and that others compounds such as peptides, which are also recognized as effective chelating agents [69], may be the main contributors to the observed activities. Research by Wang et al. [48,58] has reported the absence of clear correlations between TPC and FIC ability of macro-algal extracts.

As for ACE-inhibitory activity, a perfect correlation was observed between ACE-inhibition and peptide content (*r* = 1) and strong correlations were observed between ACE-inhibition and FIC activity (*r* = 0.822), between ACE-inhibition and FRAP (*r* = 0.932), and between ACE-inhibition and TPC (*r* = 0.883). Only the correlation between ACE-inhibition and FRSA was moderate (*r* = 0.660). These findings suggest that either some peptides, which are recognized as effective ACE-inhibitors agents or polyphenols are contributing to the ACE-inhibition observed in all FSPH fractions.

Overall results from this study suggest that ACE-inhibition and antioxidant activities observed in FSPH fractions may be due to the sum of the activities of produced peptides and released polyphenols. However, in order to better understand the contribution of peptides and polyphenols to the aforementioned bifunctional properties, a complete chemical characterization of FSPH fractions should be done in future.

## 3. Materials and Methods

### 3.1. Chemicals and Reagents

Methanol (MeOH) and acetonitrile (ACN) HPLC grade were purchased from Fluka Chemika (Steinheim, Switzerland). Ammonium sulphate, sodium carbonate (Na_2_CO_3_), sodium phosphate, sodium tetraborate decahydrate, iron (II) chloride, sodium hydroxide (NaOH), ethanol, phenol and 85% phosphoric acid were from E. Merck (Darmstadt, Germany). Acetyl chloride and isobutanol were purchased from Alltech Associates (Deerfield, IL, USA). Amino acids standard mixture and sequanal grade 6 N HCl were purchased from Pierce Chemicals (Rockford, IL, USA). Sodium chloride (NaCl), ethyl acetate, heptafluorobutyric anhydride (HFB-IBA), sodium caseinate, 2-mercaptoethanol, brilliant Blue G-250, albumin from bovine serum (BSA), potassium ferricyanide, iron (III) chloride, ferrozine, trichloroacetic acid (TCA), trizma base, zinc chloride, hippuric acid (HA), hippuryl-l-histidyl-l-leucine (HHL), Folin–Ciocalteu reagent (FCR), phloroglucinol, butylated hydroxytoluene (BHT), ethylenediaminetetraacetic acid (EDTA), 2,2-diphenyl-1-picrylhydrazyl (DPPH), hydrochloric acid (HCl), glutathione, *O*-phthaldialdehyde (OPA), *β*-mercaptoethanol, sodium dodecyl sulphate, angiotensin I-converting enzyme (ACE) from porcine kidney, bromelain (B4882), cellulase from *Aspergillus niger* (C1184), chymotrypsin (C9381), peptidase (P7500) and trypsin (T8003) were obtained from Sigma-Aldrich (St. Louis, MO, USA). Ultrafiltration membrane system and membranes were purchased from Millipore Co (Bedford, MA, USA).

### 3.2. Collection and Preparation of F. spiralis Sample

*F. spiralis* Linnaeus (Ochrophyta, Phaeophyceae) sample was collected in January 2013 from the littoral of São Miguel Island of Azores Archipelago (37°40′ N and 25°31′ W), Portugal, and a voucher specimen was prepared (voucher number AZB, SMG-13-04) and deposited in the Herbarium AZB—Ruy Telles Palhinha of the Department of Biology at the University of Azores. Within 24 h of collection, *F. spiralis* sample was first washed in seawater followed by distilled water to remove encrusting material, epiphytes and salts, and then air-dried and stored in an air-tight container in a freezer (−80 °C) for not more than 6 months until further analysis. Prior to the analytical procedures, the sample was defrosted and dried at 40–45 °C for 48 h (avoiding overheating that could lead to oxidation), and then was grounded into a fine powder of 0.5 mm particle size, re-dried at 40 °C and stored in the dark under N_2_ in a desiccator at a refrigerated temperature of 4–5 °C.

### 3.3. Extraction of Protein from F. spiralis

The protein of *F. spiralis* was extracted according to the method described by Wong et al. [70] with slight modifications. Five grams of sample powder were suspended in deionized water (1:10 *w*/*v*) to induce cell lysis by osmotic shock that facilitated subsequent protein extraction. The suspension was gently stirred overnight at 35 °C, which was found to be the optimal temperature for macro-algae protein solubility. After incubation, the suspension was centrifuged at 10,000× *g* and 4 °C for 20 min. The supernatant was collected and the pellet was re-suspended in deionized water with 0.5% 2-mercaptoethanol (*v*/*v*) being the pH adjusted to 12.0 with 1 M NaOH. The solution was gently stirred at room temperature for 2 h before centrifugation under the same conditions as above. The second supernatant was collected and combined with the first one. The combined supernatant was stirred at 4 °C and the pH was adjusted to 7.0 before precipitation with ammonium sulphate. The extraction method mentioned above was repeated three times on the residue. The combined supernatant was precipitated by slowly adding ammonium sulphate, with stirring, until 85% saturation (60 g/100 mL) and allowed to stand for 30 min. Then the solution (precipitated protein) was removed by centrifugation at 10,000× *g*, 4 °C for 20 min. The pellet obtained was dialyzed against distilled water until the total dissolved solutes of dialysate, measured by its conductivity, was similar to that of the distilled water. The retentate containing the protein concentrate of *F. spiralis* was lyophilized in a freeze-drier and stored at −20 °C until required. The protein content was determined by Bradford method [71] using BSA as the calibration standard.

### 3.4. In Vitro F. spiralis Protein Digestibility Evaluation

The in vitro digestibility of *F. spiralis* protein concentrate suspension was determined using a freshly prepared multiproteolytic enzyme solution (trypsin, chymotrypsin and peptidase), according to Paiva et al. [72], in order to reproduce the actual digestion environment in vivo. Fifty milliliters of aqueous protein suspension (6.25 mg protein/mL) in glass distilled water were adjusted to pH 8.0, while stirring in a 37 °C water bath. The multi-enzyme solution [1.6 mg/mL trypsin (10,000 BAEE units/mg protein), 3.1 mg/mL chymotrypsin (80 units/mg solid) and 1.3 mg peptidase/mL (50–100 units/g solid)] was maintained in an ice bath and adjusted to pH 8.0. Five milliliters of the multi-enzyme solution were then added to the protein suspension and the pH change in the mixture, caused by the enzymatic digestion, was measured after exactly 10 min. Sodium caseinate was used as control and the in vitro protein digestibility of the algae was expressed as a relative percentage to that of the sodium caseinate normalized at 100%.

### 3.5. Preparation of F. spiralis Protein Hydrolysate (FSPH)

The enzymatic reaction of the *F. spiralis* protein concentrate was carried out using two enzymes, cellulase followed by bromelain. One gram of protein concentrate sample was suspended and homogenized in 10 mL of ice-cold distilled water and each enzyme (substrate/enzyme ratio to 100:1, *w*/*w*) was individually added to the homogenate. For the enzymatic reaction was used cellulase at 50 °C and pH 4.5 (adjusted with 0.1 M HCl) followed by bromelain at 37 °C and pH 7.0 (adjusted with 0.5 M NaOH). The hydrolytic reaction was carried out for 20 h and after this period, the digestion solution was boiled for 10 min in order to inactivate the enzymes. The protein hydrolysate solution was centrifuged at 3000× *g* for 15 min, filtered through 0.45 μm filters, lyophilized in a freeze-drier and stored at −20 °C for further analysis.

### 3.6. Fractionation of FSPH by Ultrafiltration

The digested FSPH with cellulase and bromelain enzymes was further fractionated in a cell dialyzer system through three different ultrafiltration membranes with molecular weight cut-off (MWCO) of 1 and 3 kDa to obtain ultrafiltrates (Fr_1_ < 1 kDa, 1 kDa ≤ Fr_2_ < 3 kDa and Fr_3_ ≥ 3 kDa). All the pooled FSPH fractions were lyophilized in a freeze-drier and assayed for ACE-inhibitory and antioxidant activities. The recovery yield of the ultrafiltration fractions was calculated using the gravimetric method.

### 3.7. Protein and Peptide Contents Analysis of FSPH Fractions

The protein content was determined by Bradford method [71] using BSA as the calibration standard and its peptide content was measured by OPA method [73] using glutathione as the calibration standard, according to Ghanbari et al. [46].

### 3.8. Amino Acids Composition of FSPH Fractions

The amino acids composition of FSPH fractions was determined according to Paiva et al. [3]. The dried FSPH fractions (0.5 mg) were placed in a small reaction vials, exposed to a stream of dry nitrogen, then capped and submitted to an acid hydrolysis at 100 °C for 24 h with 100 µL 6 N HCl containing 0.1% phenol for tyrosine protection. After cooling until room temperature, the samples were evaporated under a stream of dry nitrogen and then derivatized slowly by adding the mixture of acetyl chloride:isobutanol (1.25:5 mL, *v*/*v*, the reagent was obtained by adding the acetyl chloride to isobutanol precooled to −20 °C) and heating at 100 °C for 60 min. Next, the sample vials were opened and the mixtures exposed to a stream of dry nitrogen to remove excess reagent. After cooling in an ice bath, the samples were supplemented with 200 µL of acetonitrile and 50 µL of the derivatization reagent HFB-IBA, and heated again at 100 °C for 15 min. After evaporation of the excess reagent at 115 °C under a stream of dry nitrogen and cooling to room temperature, samples were dissolved in 300 µL of ethyl acetate and an aliquot (1 µL) was used for GC analysis. The GC analysis was performed using a Bruker GC model 450-GC gas chromatograph equipped with a split/splitless injector and a flame ionization detector (FID) using a wall-coated open tubular (WCOT) fused silica AT-Amino acid capillary column (25 m × 0.53 mm i.d., 1.2 µm film thickness) from Heliflex/Alltech (San Jose, CA, USA). The temperature started at 60 °C for 3 min, programmed at a rate of 4 °C/min to 210 °C and then held at this temperature for further 20 min. The injector and detector temperatures were held constants at 260 °C and 280 °C, respectively. Helium was the carrier gas at a flow rate of 28 cm/s.

### 3.9. Total Phenolic Content (TPC) Determination of FSPH Fractions

TPC was determined according to the method of Waterhouse [74] with slight modifications. An aliquot of 100 µL of FSPH fraction (2 mg/mL) was mixed with 1500 µL of distilled water and 100 µL of 2N FCR, homogenized in a vortex for 15 s and placed in dark for 3 min. Then, 300 µL of 10% Na_2_CO_3_ (*w*/*v*) was added to the mixture, homogenized and incubated for 5 min at 50 °C. The Abs values were measured at 760 nm. A blank sample was prepared by replacing the sample with Milli-Q water. The phloroglucinol (a basic structural unit of phlorotannins) was used as a standard and the results are expressed as mg of phloroglucinol equivalents (PE) per gram of dried FSPH fraction. A calibration curve was prepared using a concentration range of 50–300 μg/mL.

### 3.10. ACE-Inhibitory Activity Determination of FSPH Fractions

The determination of ACE-inhibitory activity was performed in vitro by RP-HPLC adapted from the spectrophotometric method described by Cushman and Cheung [75] with slight modifications [3]. This method is based on the liberation of hippuric acid from hippuryl-l-histidyl-l-leucine (Hip-His-Leu) catalyzed by ACE. For the assay, 42.5 µL of the sample solution (2 mg/mL) was pre-incubated at 37 °C for 5 min with 10 µL ACE (0.6 mU/mL) enzyme. The mixture was subsequently incubated at the same temperature for 60 min with 20 µL of the substrate (5 mM HHL in 10 µM zinc chloride containing 100 mM sodium trizma base and 300 mM NaCl at pH 8.3). The reaction was terminated by adding 12.5 µL of 5 M HCl. The percentage of ACE-inhibition was determined by an HPLC system from Waters equipped with a 626 pump and 600S controller coupled to a 486 tunable UV detector. An aliquot of 20 µL from the reaction mixture was analyzed on a reverse-phase Ultrasphere ODS analytical column (25 cm × 4.6 mm i.d., 5 µm particle size) (Beckman Coulter, Miami, FL, USA) using an isocratic elution of MeOH: ACN: 0.1% HCl (25:25:50, *v*/*v*/*v*) at a constant flow-rate of 0.6 mL/min and HA and HHL were detected by UV at 228 nm. The average value from three determinations at each concentration was used to calculate the ACE-inhibition (%) rate as follows: % ACE-inhibition = [B − A/B − C] × 100, where A is the absorbance (Abs) of HA generated in the presence of ACE-inhibitor, B the Abs of HA generated without ACE-inhibitor and C the Abs of HA generated without ACE (corresponding to HHL autolysis in the course of enzymatic assay). The IC_50_ value (mg/mL) was defined as the concentration of inhibitor required to reduce the HA peak by 50% (corresponding to 50% inhibition of ACE activity). The captopril was used as a positive control for ACE-inhibition.

### 3.11. Antioxidant Activity Assays on FSPH Fractions

#### 3.11.1. DPPH Free Radical Scavenging Activity (FRSA) Assay

The FRSA of FSPH fractions was determined according to the method of Molyneux [76] with slight modifications. The FRSA of each FSPH fraction was tested by measuring their ability to quench DPPH. The DPPH, a stable free radical, is reduced changing the purple color of the DPPH radical solution to a bright yellow in the presence of antioxidants that possess hydrogen-donating or chain-breaking properties and the intensity of this can be monitored spectrophotometrically [77]. An aliquot of 250 µL of FSPH fraction with various concentrations (or BHT) was added to 250 µL of 100 mM DPPH solution. BHT was used as reference sample and a mixture without FSPH sample or BHT was used as the control. The Abs was measured at 517 nm after 30 min in the dark. The FRSA of FSPH fractions was calculated as a percentage of DPPH decoloration using the following equation: % FRSA = (1 − Abs_sample_/Abs_control_) × 100.

#### 3.11.2. Ferrous Ion-Chelating (FIC) Activity Assay

Chelating ability of FSPH fractions was determined according to the modified method of Wang et al. [58], by measuring the inhibition of the Fe^2+^–ferrozine complex formation. An aliquot of 100 µL of each FSPH fraction (concentration 2 mg/mL) was mixed with 135 µL of methanol plus 5 µL of 2 mM FeCl_2_. The reaction was initiated by the addition of 10 µL of 5 mM ferrozine. After 10 min at room temperature, the Abs was determined at 562 nm. Methanol instead of ferrozine solution was used as a sample blank, which is used for error correction because of unequal color of the sample solutions. Methanol instead of sample solution was used as a control. Results are expressed as relative iron chelating activity compared with the unchelated (without ferrozine) Fe^2+^ reaction, and EDTA was used as reference standard. A lower Abs indicated a better FIC ability. The FIC ability was calculated as follows: % FIC ability = [A_0_ − (A_1_ − A_2_)]/A_0_ × 100, where A_0_ was the Abs of the control, A_1_ was the Abs of the sample or standard and A_2_ was the Abs of the blank.

#### 3.11.3. Ferric Reducing Antioxidant Power (FRAP) Assay

The FRAP of FSPH fractions was determined according to the method of Oyaizu [78], and evaluated on the basis of their abilities to reduce Fe^3+^ complex to Fe^2+^. An increased Abs value indicates an increased reducing power of the hydrolysate fractions. Each fraction (concentration range 12.5–100 μg/mL) in methanol (0.4 mL) was mixed with 0.4 mL of 300 mM of phosphate buffer (pH 6.6) plus 0.4 mL of potassium ferricyanide (1%, *w*/*v*) and the mixture was incubated at 50 °C for 20 min. After cooling down, 0.4 mL of TCA (10%, *w*/*v*) was added, and the mixture was centrifuged at 3000× *g* for 10 min. The upper layer (1 mL) was mixed with 1 mL of deionized water plus 0.2 mL of FeCl_3_ (0.1% *w*/*v*), and the Abs was measured at 700 nm against a blank. The blank solution contained pure methanol instead of the methanolic FSPH fraction. BHT was used for comparison.

### 3.12. Statistical Analysis

All determinations were performed at least in triplicate and the results were expressed as means ± standard deviations (SD). The statistics analysis was performed using SPSS 17.0 (version 17, SPSS Inc., Chicago, IL, USA) and one-way analysis of variance test (ANOVA) was carried out to assess for any significant differences between the means. Differences between means at the 5% (*p <* 0.05) level were considered significant. Correlations between the parameters evaluated were obtained using Pearson’s correlation coefficient (*r*).

## 4. Conclusions

Algae remain a relatively untapped source of compounds with ACE-inhibitory and antioxidant activities. To the best of our knowledge, this study revealed, for the first time, that enzymatic hydrolysate fractions with the aforementioned bifunctional properties could be efficiently generated from *F. spiralis* protein hydrolyzed by the cellulase–bromelain enzymes. The fraction with MW ≥3 kDa, obtained from this hydrolysate using ultrafiltration membranes, showed significantly higher recovery yield, ACE-inhibition and ferric reducing antioxidant power than the other fractions, and also showed strong scavenging of DPPH radical and ferrous ions (Fe^2+^) chelating activity. The significant bioactivity of this fraction can be attributed to the high concentration of the potent active peptide sequences (even though the fraction contains peptides relatively large in size) and phenolic compounds (high-MW phlorotannins). Although additional research is needed on structural information of the bioactive compounds, overall results from this study indicate that the produced *F. spiralis* protein hydrolysate fractions can be promising natural sources to develop functional food ingredients for controlling hypertension and/or oxidative stress, the two major causes of cardiovascular diseases. However, in vivo studies are also needed to verify their physiological effects.

## Figures and Tables

**Table 1:** Angiotensin I-converting enzyme (ACE) inhibitory activity, recovery yield, protein and peptide contents of *F. spiralis* protein hydrolysate (FSPH) fractions obtained by ultrafiltration.

Sample	Yield (mg/g of DW FSPH)	ACE-Inhibition		Protein Content (mg/g of DW FSPH)	Peptide Content (mg/g of DW FSPH)
		Percentage	IC_50_ Value (mg/mL)		
Fr_1_ < 1 kDa	206.28	45.08 ± 0.66 ^a^	1.850 ± 0.06 ^b^	123.15 ± 2.78 ^a^	43.81 ± 2.27 ^a^
1 kDa ≤ Fr_2_ < 3 kDa	53.72	41.93 ± 1.62 ^a^	2.000 ± 0.06 ^b^	336.28 ± 4.96 ^b^	35.91 ± 1.58 ^a^
Fr_3_ ≥ 3 kDa	703.95	86.85 ± 1.89 ^b^	0.500 ± 0.03 ^a^	474.03 ± 4.44 ^b^	243.82 ± 2.9 ^b^

Values are mean ± SD (*n* = 3). Different superscript letters are significantly different (*p <* 0.05). IC_50_ value defined as the concentration which inhibits 50% of the ACE activity (tested concentration = 2 mg/mL). Captopril, used as a positive control for ACE-inhibition, showed an IC_50_ value of 0.163 ng/mL. DW, dry weight.

**Table 2:** Amino acid profiles of *F. spiralis* protein hydrolysate fractions (mg amino acids/g of DW FSPH) obtained by ultrafiltration.

Amino Acid (AA)	*F. spiralis* Protein Hydrolysate (FSPH) Fraction		
	Fr_1_ < 1 kDa	1 kDa ≤ Fr_2_ < 3 kDa	Fr_3_ ≥ 3 kDa
Alanine	tc	tc	25.34 ± 0.13
Glycine	tc	tc	22.38 ± 0.13
Valine	155.53 ± 2.25	209.37 ± 3.35	236.83 ± 4.65
Threonine	1.22 ± 0.08	1.71 ± 0.03	17.42 ± 0.13
Serine	2.75 ± 0.06	3.18 ± 0.03	19.36 ± 0.16
Leucine	2.24 ± 0.12	2.85 ± 0.05	25.35 ± 0.17
Isoleucine	1.89 ± 0.03	2.84 ± 0.06	32.55 ± 0.43
Proline	1.34 ± 0.05	1.93 ± 0.03	12.47 ± 0.11
Methionine	tc	tc	20.07 ± 0.31
Aspartic acid	3.63 ± 0.07	4.53 ± 0.10	37.53 ± 0.35
Phenylalanine	1.80 ± 0.02	1.49 ± 0.02	15.13 ± 0.15
Glutamic acid	10.40 ± 0.10	7.01 ± 0.22	46.33 ± 0.42
Lysine	15.48 ± 0.20	7.57 ± 0.11	13.82 ± 0.13
Tyrosine	tc	tc	38.17 ± 0.21
Arginine	tc	tc	tc
Histidine	tc	tc	tc
Tryptophan	ND	ND	ND
Total AA	196.28 ^a^	242.48 ^a^	562.75 ^b^
AA distribution (%)			
Hydrophobic	82.94 ^a^	90.10 ^a^	65.35 ^b^
Hydrophilic	15. 04 ^a^	7.88 ^b^	17.36 ^a^
Neutral	2.02 ^b^	2.02 ^b^	17.29 ^a^
Aromatic	0.92 ^b^	0.61 ^b^	9.47 ^a^
Branched-side chains	81.34 ^a^	88.69 ^a^	52.37 ^b^
Negatively charged	7.15 ^b^	4.76 ^c^	14.90 ^a^
Positively charged	7.89 ^a^	3.12 ^b^	2.46 ^b^

Values are mean ± SD (*n = 3*). Means with different superscripts letters in the same row differ significantly (*p* < 0.05). Hydrophobic AA (Ala, Val, Leu, Ile, Pro, Met, Phe). Hydrophilic AA (Asp, Glu, Lys, Arg, His). Neutral AA (Gly, Thr, Ser, Tyr). Aromatic AA (Phe, Tyr). Branched-side chains AA (Val, Leu, Ile). Negatively charged AA (Asp, Glu). Positively charged AA (Lys, Arg, His). Tryptophan is not detected in this methodology. DW, dry weight; ND, not detected; tc, traces.

**Table 3:** Correlation matrix of the studied parameters (Pearson correlations coefficients).

	FRSA	FIC Activity	FRAP	Total Phenolic	ACE-Inhibition	Peptide Content
FRSA	1	-	-	-	-	-
FIC activity	0.890	1		-	-	-
FRAP	0.760	0.973	1	-	-	-
Total phenolic	0.003	0.458	0.652	1	-	-
ACE-inhibition	0.660	0.822	0.932	0.883	1	-
Peptide content	0.446	0.805	0.921	0.896	1	1

FRSA, free radical scavenging activity; FIC, ferrous ions (Fe^2+^) chelating; FRAP, ferric reducing antioxidant power; ACE, angiotensin I-converting enzyme.

## References

[1] Lordan S., Ross R.P., Stanton C. (2011). Marine bioactives as functional food ingredients: Potential to reduce the incidence of chronic diseases. Mar. Drugs.

[2] Hata Y., Nakajima K., Uchida J.I., Hidaka H., Nakano T. (2001). Clinical effects of brown seaweed, *Undaria pinnatifida* (*wakame*), on blood pressure in hypertensive subjects. J. Clin. Biochem. Nutr..

[3] Paiva L., Lima E., Neto A.I., Baptista J. (2016). Isolation and characterization of angiotensin I-converting enzyme (ACE) inhibitory peptides from *Ulva rigida* C. Agardh protein hydrolysate. J. Funct. Foods.

[4] Soffer R.L. (1976). Angiotensin-converting enzyme and the regulation of vasoactive peptides. Annu. Rev. Biochem..

[5] Zhang Y., Lee E.T., Devereux R.B., Yeh J., Best L.G., Fabsitz R.R., Howard B.V. (2006). Prehypertension, diabetes, and cardiovascular disease risk in a population-based sample: The strong heart study. Hypertension.

[6] Andrews P.R., Carson J.M., Caselli A., Spark M.J., Woods R. (1985). Conformational analysis and active site modelling of angiotensin-converting enzyme inhibitors. J. Med. Chem..

[7] Suetsuna K. (1998). Purification and identification of angiotensin I-converting enzyme inhibitors from the red alga *Porphyra yezoensis*. J. Mar. Biotechnol..

[8] Sato M., Hosokawa T., Yamaguchi T., Nakano T., Muramoto K., Kahara T., Funayama K., Kobayashi A., Nakano T. (2002). Angiotensin I-converting enzyme inhibitory peptides derived from Wakame (*Undaria pinnatifida*) and their antihypertensive effect in spontaneously hypertensive rats. J. Agric. Food Chem..

[9] Paiva L., Lima E., Neto A.I., Baptista J. (2016). Angiotensin I-converting enzyme (ACE) inhibitory activity of *Fucus spiralis* macroalgae and influence of the extracts storage temperature: A short report. J. Pharm. Biomed. Anal..

[10] Jung H.A., Hyun S.K., Kim H.R., Choi J.S. (2006). Angiotensin-converting enzyme I inhibitory activity of phlorotannins from *Ecklonia stolonifera*. Fish. Sci..

[11] Wijesekara I., Kim S.-K. (2010). Angiotensin-I-converting enzyme (ACE) inhibitors from marine resources: Prospects in the pharmaceutical industry. Mar. Drugs.

[12] Wijesinghe W.A.J.P., Ko S.-C., Jeon Y.-J. (2011). Effect of phlorotannins isolated from *Ecklonia cava* on angiotensin I-converting enzyme (ACE) inhibitory activity. Nutr. Res. Pract..

[13] Olivares-Molina A., Fernández K. (2016). Comparison of different extraction techniques for obtaining extracts from brown seaweeds and their potential effects as angiotensin I-converting enzyme (ACE) inhibitors. J. Appl. Phycol..

[14] Catarino M.D., Silva A.M.S., Cardoso S.M. (2017). Fucaceae: A source of bioactive phlorotannins. Int. J. Mol. Sci..

[15] Harnedy P.A., FitzGerald R.J. (2011). Bioactive proteins, peptides and amino acids from macroalgae. J. Phycol..

[16] Samarakoon K., Jeon Y.-J. (2012). Bio-functionalities of proteins derived from marine algae—A review. Food Res. Int..

[17] Cian R.E., Alaiz M., Vioque J., Drago S.R. (2013). Enzyme proteolysis enhanced extraction of ACE inhibitory and antioxidant compounds (peptides and polyphenols) from *Porphyra columbina* residual cake. J. Appl. Phycol..

[18] Oroian M., Escriche I. (2015). Antioxidants: Characterization, natural sources, extraction and analysis. Food Res. Int..

[19] Korhonen H., Pihlanto A. (2003). Food-derived bioactive peptides—Opportunities for designing future foods. Curr. Pharm. Des..

[20] Wijesinghe W.A., Jeon Y.-J. (2012). Enzyme-assistant extraction (EAE) of bioactive components: A useful approach for recovery of industrially important metabolites from seaweeds: A review. Fitoterapia.

[21] Neto A.I., Brotas V., Azevedo J.M.N., Patarra R.F., Álvaro N.M.V., Gameiro C., Prestes A.C.L., Nogueira E.M. (2009). Qualidade de Águas Costeiras do Grupo Oriental do Arquipélago dos Açores e Proposta de Monitorização.

[22] Neto A.I., Tittley I., Raposeiro P.M. (2005). Flora Marinha do Litoral dos Açores. Rocky Shore Marine Flora of the Azores.

[23] Paiva L., Lima E., Patarra R.F., Neto A.I., Baptista J. (2014). Edible Azorean macroalgae as source of rich nutrients with impact on human health. Food Chem..

[24] Cerantola S., Breton F., Ar Gall E., Deslandes E. (2006). Co-occurrence and antioxidant activities of fucol and fucophlorethol classes of polymeric phenols in *Fucus spiralis*. Bot. Mar..

[25] Tierney M.S., Smyth T.J., Rai D.K., Soler-Vila A., Croft A.K., Brunton N. (2013). Enrichment of polyphenol contents and antioxidant activities of Irish brown macroalgae using food-friendly techniques based on polarity and molecular size. Food Chem..

[26] Pinteus S., Silva J., Alves C., Horta A., Fino N., Inês Rodrigues A., Mendes S., Pedrosa R. (2017). Cytoprotective effect of seaweeds with high antioxidant activity from the Peniche coast (Portugal). Food Chem..

[27] Fleurence J. (1999). Seaweed proteins: Biochemical nutritional aspects and potential uses. Trends Food Sci. Technol..

[28] Quiroz-Castañeda R.E., Folch-Mallol J.L., Chandel A.K., da Silva S.S. (2013). Hydrolysis of biomass mediated by cellulases for the production of sugars. Sustainable Degradation of Lignocellulosic Biomass-Techniques, Applications and Commercialization.

[29] Paiva L., Lima E., Neto A.I., Baptista J. (2015). Screening for angiotensin I-converting enzyme (ACE) inhibitory activity of enzymatic hydrolysates obtained from Azorean macroalgae. Arquipél. Life Mar. Sci..

[30] Elavarasan K., Kumar V.N., Shamasundar B.A. (2014). Antioxidant and functional properties of fish protein hydrolysates from fresh water carp (*Catla catla*) as influenced by the nature of enzyme. J. Food Process. Preserv..

[31] Gajanan P.G., Elavarasan K., Shamasundar B.A. (2016). Bioactive and functional properties of protein hydrolysates from fish frame processing waste using plant proteases. Environ. Sci. Pollut. Res..

[32] Auwal S.M., Zarei M., Abdul-Hamid A., Saari N. (2017). Optimization of bromelain-aided production of angiotensin I-converting enzyme inhibitory hydrolysates from stone fish using response surface methodology. Mar. Drugs.

[33] Chalé F.G.H., Ruiz J.C.R., Fernández J.J.A., Ancona D.A.B., Campos M.R.S. (2014). ACE inhibitory, hypotensive and antioxidant peptide fractions from *Mucuna pruriens* proteins. Process Biochem..

[34] Udenigwe C.C., Aluko R.E. (2011). Chemometric analysis of the amino acid requirements of antioxidant food protein hydrolysates. Int. J. Mol. Sci..

[35] Fleurence J., Yada R.Y. (2004). Seaweed proteins. Proteins in Food Processing.

[36] Ge Y., Sun A., Ni Y., Cai T. (2001). Study and development of a defatted wheat germ nutritive noodle. Eur. Food Res. Technol..

[37] Athukorala Y., Jeon Y.J. (2005). Screening for angiotensin I-converting enzyme inhibitory activity of *Ecklonia cava*. J. Food Sci. Nutr..

[38] Tierney M.S., Soler-vila A., Croft A.K., Hayes M. (2013). Antioxidant activity of the brown macroalgae *Fucus spiralis* Linnaeus harvested from the west coast of Ireland. Curr. Res. J. Biol. Sci..

[39] Wang T., Jónsdóttir R., Liu H., Gu L., Kristinsson H.G., Raghavan S., Ólafsdóttir G. (2012). Antioxidant capacities of phlorotannins extracted from the brown algae *Fucus vesiculosus*. J. Agric. Food Chem..

[40] Steevensz A.J., MacKinnon S.L., Hankinson R., Craft C., Connan S., Stengel D.B., Melanson J.E. (2012). Profiling phlorotannins in brown macroalgae by liquid chromatography-high resolution mass spectrometry. Phytochem. Anal..

[41] Ferreres F., Lopes G., Gil-Izquierdo A., Andrade P.B., Sousa C., Mouga T., Valentão P. (2012). Phlorotannin extracts from Fucales characterized by HPLC-DAD-ESI-MS^*n*^: Approaches to hyaluronidase inhibitory capacity and antioxidant properties. Mar. Drugs.

[42] Zhao Y., Li B., Dong S., Liu Z., Zhao X., Wang J., Zeng M. (2009). A novel ACE inhibitory peptide isolated from *Acaudina molpadioidea* hydrolysate. Peptides.

[43] Hong L.G., Wei L.G., Liu H., Hui S.Y. (2005). Mung-bean protein hydrolysates obtained with Alcalase exhibit angiotensin I-converting enzyme inhibitory activity. Food Sci. Technol. Int..

[44] Wilson J., Hayes M., Carney B. (2011). Angiotensin-I-converting enzyme and prolyl endopeptidase inhibitory peptides from natural sources with a focus on marine processing by-products. Food Chem..

[45] Segura-Campos M.R., Peralta-González F., Castellanos-Ruelas A., Chel-Guerrero L.A., Betancur-Ancona D.A. (2013). Effect of *Jatropha curcas* peptide fractions on the angiotensin I-converting enzyme inhibitory activity. Biomed. Res. Int..

[46] Ghanbari R., Zarei M., Ebrahimpour A., Abdul-Hamid A., Ismail A., Saari N. (2015). Angiotensin-I converting enzyme (ACE) inhibitory and anti-oxidant activities of sea cucumber (*Actinopyga lecanora*) hydrolysates. Int. J. Mol. Sci..

[47] Liu J.-C., Hsu F.-L., Tsai J.-C., Chan P., Liu J.Y., Thomas G.N., Tomlinson B., Lo M.-Y., Lin J.-Y. (2003). Antihypertensive effects of tannins isolated from traditional Chinese herbs as non-specific inhibitors of angiotensin converting enzyme. Life Sci..

[48] Wang T., Jónsdóttir R., Kristinsson H.G., Hreggvidsson G.O., Jónsson J.O., Thorkelsson G., Ólafsdóttir G. (2010). Enzyme-enhanced extraction of antioxidant ingredients from red algae *Palmaria palmata*. LWT—Food Sci. Technol..

[49] Fan J., He J., Zhuang Y., Sun L. (2012). Purification and identification of antioxidant peptides from enzymatic hydrolysates of Tilapia (*Oreochromis niloticus*) frame protein. Molecules.

[50] Udenigwe C.C., Lu Y.-L., Han C.-H., Hou W.-C., Aluko R.E. (2009). Flaxseed protein-derived peptide fractions: Antioxidant properties and inhibition of lipopolysaccharide-induced nitric oxide production in murine macrophages. Food Chem..

[51] Chen H.-M., Muramoto K., Yamauchi F., Fujimoto K., Nokihara K. (1998). Antioxidative properties of histidine-containing peptides designed from peptide fragments found in the digests of a soybean protein. J. Agric. Food. Chem..

[52] Guo H., Kouzuma Y., Yonekura M. (2009). Structures and properties of antioxidative peptides derived from royal jelly protein. Food Chem..

[53] Rajapakse N., Mendis E., Jung W.-K., Je J.-Y., Kim S.-K. (2005). Purification of a radical scavenging peptide from fermented mussel sauce and its antioxidant properties. Food. Res. Int..

[54] Nimalaratne C., Bandara N., Wu J. (2015). Purification and characterization of antioxidant peptides from enzymatically hydrolyzed chicken egg white. Food Chem..

[55] Alemán A., Pérez-Santín E., Bordenave-Juchereau S., Arnaudin I., Gómez-Guillén M.C., Montero P. (2011). Squid gelatin hydrolysates with antihypertensive, anticancer and antioxidant activity. Food Res. Int..

[56] Heffernan N., Smyth T.J., Soler-Villa A., Fitzgerald R.J., Brunton N.P. (2015). Phenolic content and antioxidant activity of fractions obtained from selected Irish macroalgae species (*Laminaria digitata*, *Fucus serratus*, *Gracilaria gracilis* and *Codium fragile*). J. Appl. Phycol..

[57] Kim K.N., Heo S.-J., Song C.B., Lee J., Heo M.S., Yeo I.K., Kang K., Hyun J.W., Jeon Y.-J. (2006). Protective effect of *Ecklonia cava* enzymatic extracts on hydrogen peroxide-induced cell damage. Process Biochem..

[58] Wang T., Jónsdóttir R., Ólafsdóttir G. (2009). Total phenolic compounds, radical scavenging and metal chelation of extracts from Icelandic seaweeds. Food Chem..

[59] Torres-Fuentes C., Alaiz M., Vioque J. (2011). Affinity purification and characterisation of chelating peptides from chickpea protein hydrolysates. Food Chem..

[60] Girgih A.T., Udenigwe C.C., Aluko R.E. (2011). In vitro antioxidant properties of hemp seed (*Cannabis sativa* L.) protein hydrolysate fractions. J. Am. Oil Chem. Soc..

[61] Senevirathne M., Kim S.-H., Siriwardhana N., Ha J.-H., Lee K.-W., Jeon Y.-J. (2006). Antioxidant potential of *Ecklonia cava* on reactive oxygen species scavenging, metal chelating, reducing power and lipid peroxidation inhibition. Revista de Agaroquimica y Tecnologia de Alimentos.

[62] Santoso J., Yoshie-Stark Y., Suzuki T. (2004). Anti-oxidant activity of methanol extracts from Indonesian seaweeds in an oil emulsion model. Fish. Sci..

[63] Duh P.-D. (1998). Antioxidant activity of burdock (*Arctium lappa* Linné): It’s scavenging effect on free radical and active oxygen. J. Am. Oil Chem. Soc..

[64] Chandini S.-K., Ganesan P., Bhaskar N. (2008). In vitro antioxidant activities of three selected brown seaweeds of India. Food Chem..

[65] Ganesan P., Kumar C.S., Bhaskar N. (2008). Antioxidant properties of methanol extract and its solvent fractions obtained from selected Indian red seaweeds. Bioresour. Technol..

[66] Zubia M., Fabre M.S., Kerjean V., Lann K.L., Stiger-Pouvreau V., Fauchon M., Deslandes E. (2009). Antioxidant and antitumoural activities of some Phaeophyta from Brittany coasts. Food Chem..

[67] Vinayak R.C., Sabu A.S., Chatterji A. (2011). Bio-prospecting of a few brown seaweeds for their cytotoxic and antioxidant activities. Evid. Based Complement. Altern. Med..

[68] Babbar N., Oberoi H.S., Uppal D.S., Patil R.T. (2011). Total phenolic content and antioxidant capacity of extracts obtained from six important fruit residues. Food Res. Int..

[69] Pal R., Rai J.P. (2010). Phytochelatins: Peptides involved in heavy metal detoxification. Appl. Biochem. Biotechnol..

[70] Wong K.H., Cheung P.C.K., Ang P.O. (2004). Nutritional evaluation of protein concentrates isolated from two red seaweeds: *Hypnea charoides* and *Hypnea japonica* in growing rats. Hydrobiologia.

[71] Bradford M.M. (1976). A rapid and sensitive method for the quantitation of microgram quantities of protein utilizing the principle of protein-dye binding. Anal. Biochem..

[72] Paiva L., Lima E., Neto A.I., Massimo M., Baptista J. (2017). Nutritional and functional bioactivity value of selected Azorean macroalgae: *Ulva compressa*, *Ulva rigida*, *Gelidium microdon*, and *Pterocladiella capillacea*. J. Food Sci..

[73] Church F.C., Swaisgood H.E., Porter D.H., Catignani G.L. (1983). Spectrophotometric assay using *O*-phthaldialdehyde for determination of proteolysis in milk and isolated milk proteins. J. Dairy Sci..

[74] Waterhouse A.L., Wrolstad R.E. (2002). Determination of total phenolics. Current Protocols in Food Analytical Chemistry.

[75] Cushman D.W., Cheung H.S. (1971). Spectrophotometric assay and properties of the angiotensin-converting enzyme of rabbit lung. Biochem. Pharmacol..

[76] Molyneux P. (2004). The use of the stable free radical diphenylpicrylhydrazyl (DPPH) for estimating antioxidant activity. Songklanakarin J. Sci. Technol..

[77] Corrêa A.P., Daroit D.J., Coelho J., Meira S.M., Lopes F.C., Risso P.H., Brandelli A. (2011). Antioxidant, antihypertensive and antimicrobial properties of ovine milk caseinate hydrolyzed with a microbial protease. J. Sci. Food Agric..

[78] Oyaizu M. (1986). Studies on products of browning reactions: Antioxidative activities of products of browning reaction prepared from glucosamine. Jpn. J. Nutr..

